# Palmitoyltransferase ZDHHC3 Aggravates Nonalcoholic Steatohepatitis by Targeting *S*‐Palmitoylated IRHOM2

**DOI:** 10.1002/advs.202308024

**Published:** 2023-12-28

**Authors:** Minxuan Xu, Jun Tan, Liancai Zhu, Chenxu Ge, Yi Zhang, Fufeng Gao, Xianling Dai, Qin Kuang, Jie Chai, Benkui Zou, Bochu Wang

*Adv. Sci*. **2023**, *10*, 2302130

https://doi.org/10.1002/advs.202302130

In the original published article, some information is misleading and missing in the acknowledgements section. The updated acknowledgement section is:

Acknowledgements

This work was supported by 1) National Natural Science Foundation of China (NSFC Grant No.: 82200652); 2) School‐level Research Program of Chongqing University of Education (Grant No.: KY201710B & 17GZKP01); 3) Advanced Programs of Post‐doctor of Chongqing (Grant No.: 2017LY39); 4) Science and Technology Research Program of Chongqing Education Commission of China (Grant No.: KJQN201901608, KJQN201901615, KJ1601402, KJZD‐K202001603); 5) Children's Research Institute of National Center for Schooling Development Programme and Chongqing University of Education (Grant No.: CSDP19FSO1108); 6) Chongqing Professional Talents Plan for Innovation and Entrepreneurship Demonstration Team (CQCY201903258, cstc2021ycjh‐bgzxm0202); 7) Natural Science Foundation of Chongqing, China (CSTB2022NSCQ‐MSX0947), and 8) Chongqing Entrepreneurship and Innovation Support Program for Overseas Returnees (Grant No.: CX2023011).

The authors apologize for any inconvenience caused.

